# Noninvasive Tests for Liver Fibrosis Predict Postoperative Complications After Resection of Colorectal Liver Metastases

**DOI:** 10.1245/s10434-026-19338-1

**Published:** 2026-03-25

**Authors:** Christopher Tuffs, Oliver Marg, Daniar Amin, Tamal Sarkar, Christopher Schramm, Alen Kosovic, Jonathan M. Harnoss, Christoph Kahlert, Mohammed Al-Saeedi, Martin Reichert, Andreas Hecker, Elke Roeb, Martin Schneider, Moritz J. Strowitzki

**Affiliations:** 1 Justus-Liebig-University Giessen, University Hospital Giessen, Department of General, Visceral, Thoracic and Transplantation Surgery, Giessen, Germany; 2 Department of General, Visceral, and Transplantation Surgery, University Hospital Heidelberg, Heidelberg, Germany; 3 Justus-Liebig-University of Giessen, University Hospital Giessen, Department of Gastroenterology, Giessen, Germany

**Keywords:** Colorectal liver metastases, Liver resection, Liver fibrosis, Non-invasive tests, Postoperative complications

## Abstract

**Background:**

Colorectal liver metastases (CRLM) occur in up to 50% of patients who suffer from colorectal cancer. While liver resection of CRLM remains the only curative treatment option, postoperative complications are common and mitigate the beneficial effects of CRLM resection on quality of life and survival in these patients. Liver fibrosis is a known risk factor for surgical morbidity and mortality after liver surgery. However, preoperative detection of liver fibrosis remains challenging. Noninvasive tests facilitate the diagnosis of liver fibrosis without the need for liver biopsies and histological grading. We thus analyzed the significance of noninvasive tests for liver fibrosis, including FIB-4 index and SAFE score, to predict adverse short-term outcome after CRLM resection.

**Methods:**

A retrospective analysis of 107 patients who underwent surgery for CRLM at the Department for General, Visceral, Thoracic, and Transplantation Surgery of the University Hospital Giessen was performed to assess the correlation between suggested liver fibrosis as defined by noninvasive tests and short-term outcome.

**Results:**

A high FIB-4 index or SAFE score, predictive of liver fibrosis, was associated with increased incidence and severity of postoperative complications. These results were validated and confirmed in an external, independent patient cohort of 277 patients who underwent liver resection owing to CRLM at the Department for General, Visceral, and Transplantation Surgery of the University Hospital Heidelberg.

**Conclusion:**

Our findings provide a rationale for preoperative assessment of the FIB-4 index and SAFE score, as indicators of liver fibrosis, to identify CRLM patients at higher risk for postoperative complications.

**Supplementary Information:**

The online version contains supplementary material available at 10.1245/s10434-026-19338-1.

Liver metastases (CRLM) occur in up to 50% of patients with colorectal cancer and significantly shorten the overall survival of these patients.^49^ Despite developments in perioperative chemotherapy regimens and stereotactic radiotherapy,^9,10^ surgical resection of CRLM remains the only curative treatment option.^27^ Refinement of surgical techniques^20,41,44,47^ and advancements in perioperative management^22,64^ have improved operative safety and short-term outcome in patients undergoing surgery for CRLM. However, perioperative morbidity and mortality remains high,^15,17,50^ especially in multimorbid or frail patients.^21,30^

Liver fibrosis (LF) is defined as scarring of the liver tissue due to local chronic inflammation,^23^ leading to loss of liver function and increasing the risk for primary tumors^25,40^ as well as metastasis^24,29^ within the liver. Underlying diseases include alcohol-related liver disease (ALD),^58^ viral hepatitis,^3^ and metabolic dysfunction-associated steatotic liver disease (MASLD).^51^ Systemic chemotherapy, which is part of multimodal treatment for CRLM in up to 85% of cases,^36^ also contributes to LF.^75^ Even in subclinical stages of LF, overall survival of patients is impaired.^5,18,65^ Liver fibrosis is a known risk factor for surgical complications,^19,39^ worsening short- and long-term outcomes.^35^ However, diagnosis of subclinical stages of LF without transdermal biopsy remains a challenge; routine blood work and imaging from patients^38^ do not provide accurate information about the functional capacity of their liver. The Child-Pugh score^16^ and the model for end-stage liver disease (MELD) score^26^ are widely used methods to assess the severity of end-stage liver disease and, with that, the associated probability of dying without a liver transplant. However, both scores perform poorly in screening for subclinical LF and assessing the risk for postoperative complications in patients undergoing major liver resection due to primary tumors.^69,70^ As a result, reliable preoperative assessment of LF for patients undergoing surgery for CRLM is rarely performed and stratification tools to identify patients at risk for postoperative complications are rare.

Several noninvasive tests (NITs) have been developed to evaluate the patient-specific risk for the presence of LF,^4,31,59,60^ combining various clinical parameters and biochemical liver values. Established scores include the Fibrosis-4 (FIB-4) index, the Aspartate Aminotransferase to Platelet Ratio Index (APRI), the NAFLD fibrosis score (NFS), and the Steatosis-Associated Fibrosis Estimator (SAFE) score. The NFS and SAFE were evaluated in a cohort of MASLD patients, who now represent the largest group of chronic liver disease patients in Europe and worldwide.^7,43,59^

Moreover, NIT values associated with LF risk correlate with impaired long-term survival in patients with colorectal cancer or CRLM.^2,33^ Moreover, high NIT values indicate impaired short-term outcomes in patients undergoing resection of primary liver tumors.^67,72^ Nevertheless, the predictive value of NITs (in particular of the novel SAFE score) to identify patients at risk for an unfavorable short-term outcome after CRLM resection is unknown. We thus compared the performance of several recognized NITs, including the recently established SAFE score to predict postoperative complications in patients undergoing CRLM resection.

## Patients and Methods

The study was approved by the Ethics Committee of the Justus Liebig University Giessen (approval number: G216/15 with the amendment from November 27, 2023) and the Ethics Committee of the Ruprecht Karl University Heidelberg (S-649/2012) and performed under compliance with the Helsinki Declaration of 1975 (as revised in 1996). All patients were treated according to the latest national and international guidelines.

### Patient Selection Criteria

**Fig. 1:**
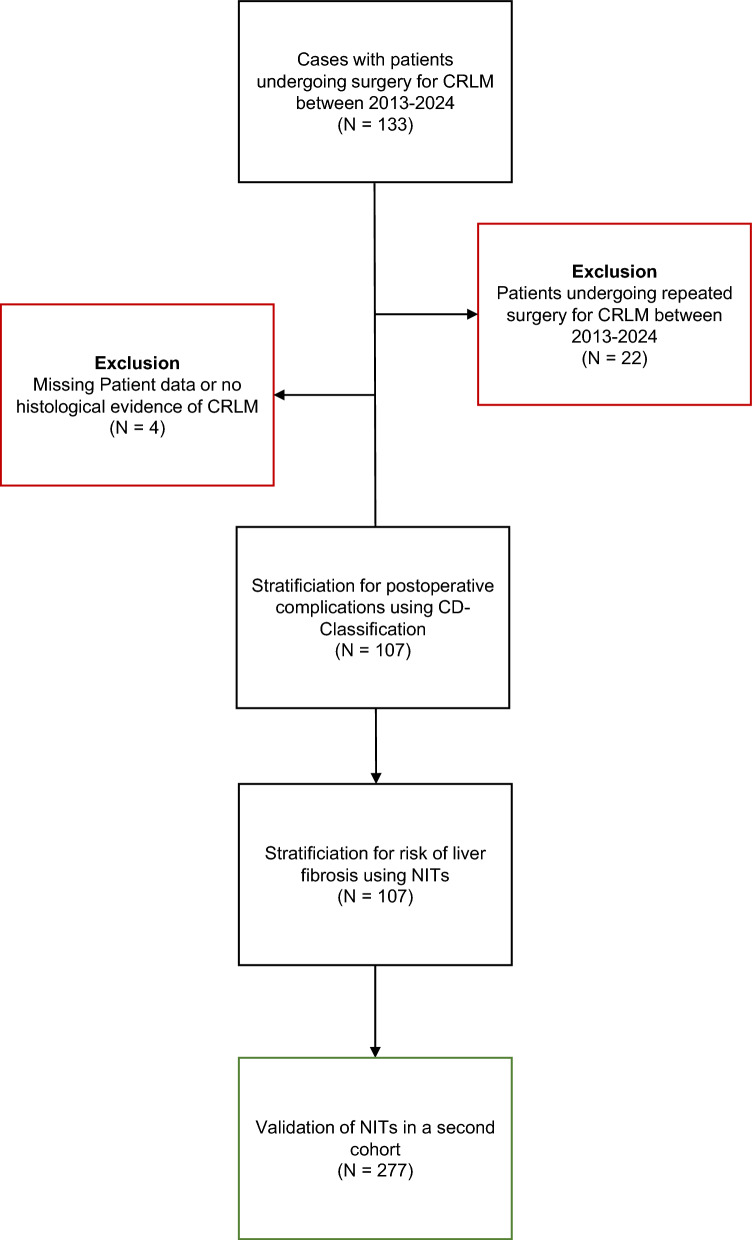
A retrospective analysis of patients undergoing surgery for CRLM at the University Hospital Giessen between 2013 and 2024 was performed. A total of 133 patients who underwent surgery were identified. Analysis of patients who underwent multiple hepatectomies was limited to their first stay and patients were excluded for whom a complete dataset was missing. The remaining 107 cases were defined as the first cohort and stratified for postoperative complications and the presence of liver fibrosis using noninvasive tests. Similarly, 277 patients were identified and analyzed in an independent second cohort to validate findings from analyses in the first cohort

Data of all patients, who underwent surgery for CRLM between 2013 and 2024 at either the Department of General, Visceral, Thoracic, and Transplantation Surgery of the University Hospital Giessen or between 2001 and 2009 at the Department of General, Visceral, and Transplantation Surgery of the University Hospital Heidelberg, were documented via a prospective database and retrospectively analysed (Fig. 1). The patient cohort from Giessen was defined as the first cohort, whereas the validation cohort from Heidelberg was defined as the second cohort. Inclusion criteria were 1) The patient was diagnosed with histologically confirmed CRLM; 2) The patient underwent liver resection due to CRLM; 3) Completeness of clinical data of the patient. Individual cases were excluded if 1) the patient underwent repeated resection for CRLM or 2) there was missing clinical data.

### Surgery

Surgeries performed in the first cohort followed the local standards of the Department of General, Visceral, Thoracic and Transplantation Surgery of the Giessen University Hospital for diagnostic workup and perioperative management. Most surgeries were performed openly by using a median laparotomy. Transection of hepatic parenchyma was frequently conducted by using ultrasonic dissection or clamp-crushing with a bipolar device and in some cases as a formal stapler hepatectomy. For anatomical resections, inflow and outflow of the targeted liver segments were controlled by dividing the corresponding branches of the hepatic artery, portal vein, and hepatic vein, either intra- or extrahepatically. In contrast, vascular control was not routinely applied during non-anatomical resections. Surgical technique for the second cohort has been described elsewhere.^56,57,62,63^ Briefly, most surgeries were performed openly using a median laparotomy and hepatectomy was mainly performed via stapler hepatectomy or using the Cavitron Ultrasonic Surgical Aspirator.

### Study Variables and Definitions

Identification and stratification of postoperative complications was conducted according to Clavien-Dindo classification.^13^ According to the International Study Group of Liver Surgery posthepatectomy liver failure was defined as concordant increase of total bilirubin and international normalized ratio on or after day 5 following liver surgery.^45^ Posthepatectomy haemorrhage was defined as a drop of haemoglobin level of 3 g/dl after surgery compared with postoperative baseline level.^46^ Major hepatectomy was defined as a formal resection of three or more liver segments^42^ The Child-Pugh score was determined according to previous publications^16^ and graded as A (5-6 points), B (7-9 points), or C (10-15 points). Acute kidney injury (AKI) was defined according to KDIGO criteria.^28^

### Calculation of Noninvasive Tests and Definition of Liver Fibrosis

Noninvasive tests were calculated by using the following formulas:

$${\boldsymbol{N}}{\boldsymbol{F}}{\boldsymbol{S}}= -1.675+0.037*age \left(years\right)+0.094*BMI \left(\frac{kg}{m^2}\right)+1.13 *diabetes \left(yes=1, no=0 \right)+0.99*\frac{AST}{ALT}ratio-0.013*\text{platelet count} \left(*\frac{{10}^{9}}{L}\right)-0.66*albumin (\frac{g}{dl})$$

$${\boldsymbol{F}}{\boldsymbol{I}}{\boldsymbol{B}}-4=\frac{age \left(years\right)*AST (\frac{U}{L})}{\text{platelet count} \left(\frac{{10}^{9}}{L}\right)* \sqrt{ALT (\frac{U}{L})}}$$

$${\boldsymbol{A}}{\boldsymbol{P}}{\boldsymbol{R}}{\boldsymbol{I}}=\frac{\frac{AST (\frac{U}{L})}{40 (\frac{U}{L})}}{\text{platelet count} (\frac{{10}^{9}}{L})} * 100$$

$${\boldsymbol{S}}{\boldsymbol{A}}{\boldsymbol{F}}{\boldsymbol{E}}=2.97*age+5.99*BMI \left(if \,BMI\,\ge 40\, it\, was\, set\, to\, 40\right)+62.85*diabetes \left(0 \,if \,absent,\, 1 \,if \,present\right)+154.85*Ln\left(AST\right)-58.23*Ln\left(ALT\right)+195. 48*Ln\left(globulin \left(\frac{g}{dl}\right)\right)-141.61* Ln (platelets \left(\frac{{10}^{9}}{\mu l}\right)-75$$

Liver fibrosis was defined by using established cutoff values for the respective NIT, indicating intermediate or high risk for LF: APRI ≥ 0.5;^31^ FIB-4 ≥ 1.45;^60^ NFS ≥ 0.675;^4^ SAFE ≥ 100.^59^

### Statistics

Continuous variables were depicted as the median ± standard deviation. Statistical differences in continuous variables of two groups were evaluated using either the two-tailed Student´s *t-*test or Mann-Whitney *U* test. Categorical variables were expressed as number of patients and frequency in percentages. Statistical differences were analysed using the chi-squared or Fisher´s exact test. Univariate and multivariate logistic regression analysis was conducted to determine independent factors associated with postoperative complications. All statistical analyses were conducted by using GraphPad Prism 7.00 (GraphPad Software, San Diego, CA, Version 7) or RStudio (Posit PBC, Boston, MA, Posit Software, Version 2024.09.1+394). *p* < 0.05 was considered significant.

## Results

In total, data for 133 cases for patients who underwent surgery for CRLM at the Surgical Department of the University Hospital Giessen were screened and considered for inclusion into the first cohort. Of 133 patients, 26 patients underwent repeated resection or showed missing clinical data and were thus excluded from further analysis. The remaining 107 patients were included into the study and defined as the first cohort (Fig. 1).

### Baseline Characteristics of All Patients from the First Cohort

Preoperative and intraoperative data of the included 107 patients who underwent surgery for CRLM are described in Table 1. The median age was 63 years. Patients were predominantly male and were neither under- nor overweight, as determined by body mass index. More than half of the patients displayed a low perioperative risk profile as defined by ASA score < 3. Almost 18% of patients were diagnosed with type 2 diabetes mellitus, and 46% of patients had cardiovascular diseases, including arterial hypertension and/or atrial fibrillation. Regarding liver function, neither median albumin, bilirubin, nor quick values were below or above a pathological threshold, resulting in 3% of enrolled patients having a Child-Pugh score grade B. Pathological evaluation of resected liver specimens revealed presence of steatosis (fat vacuoles in ≥ 5% of hepatocytes) in 33% of the patients. Most patients showed advanced tumor stage with positive lymph node status and CRLM in both liver lobes; 48% of patients received neoadjuvant (radio-) chemotherapy. The majority of operations were conducted openly and as minor liver resections. Simultaneous resection of the primary tumor was performed in 41% of the cases.

**Table 1:** Characteristics of all UICC stage 4 CRC patients

Variable		*N* (= 107) or median	(%) or SD
Gender	Female	37	(34.58%)
	Male	70	(65.42%)
Age at operation		63.40	11.67
BMI (kg/m^2^)		25.00	7.48
ASA score	I	37	(34.58%)
	II	41	(38.32%)
	≥ III	29	(27.10%)
Diabetes mellitus	Yes	19	(17.76%)
	No	88	(82.24%)
Cardiovascular disease	Yes	49	(45.79%)
	No	58	(54.21%)
Known chronic liver disease	Yes	0	(0%)
	No	107	(100%)
Child-Pugh grade	A	104	(97.19%)
	B	3	(2.81%)
Steatosis	Yes	35	(32.71%)
	No	72	(67.29%)
Albumin (g/l)		43.60	4.96
Total Bilirubin (mg/dl)		0.60	0.36
Quick (%)		102.00	20.33
Platelets (giga/l)		252.00	91.54
NFS		-1.44	1.84
FIB-4		1.48	0.86
APRI		0.24	0.22
SAFE		38.85	116.63
T	1–2	13	(12.15%)
	3–4	94	(87.85%)
N	0	37	(34.58%)
	1	44	(41.12%)
	2	26	(24.30%)
Localization of metastases	Left lobe	14	(13.08%)
	Right lobe	34	(31.78%)
	Both lobes	59	(55.14%)
Neoadjuvant treatment	No	55	(51.40%)
	Chemotherapy	44	(41.12%)
	Radiochemotherapy	8	(7.48%)
Type of liver resection	Minor	77	(71.96%)
	Major	30	(28.04%)
Surgical approach	Open	83	(77.57%)
	Laparoscopic	24	(22.43%)
Simultaneous resection of primary tumor	Yes	44	(41.12%)
	No	63	(58.88%)
Length of surgery (min)		233.00	129.81
Intraoperative blood loss (ml)		600.00	692.48
Intraoperative blood transfusion	Yes	32	(29.91%)
	No	75	(70.09%)

Data reported as n (%) or median with standard deviation. *ASA* American Society of Anesthesiologists; *APRI* AST-Platelet Ratio-index; *BMI* body mass index; *FIB-4* Fibrosis-4 Index; *NFS* NAFLD fibrosis score; *SAFE* steatosis-associated fibrosis estimator score

### Baseline Characteristics of Patients with or without Postoperative Complications

In total, 52% of the patients (56/107) developed postoperative complications (Table 2). Patients with postoperative complications had a non-significantly higher body mass index than patients without complications. Presence of comorbidities including type 2 diabetes mellitus, and cardiovascular diseases did not differ significantly between both groups (Table 2). None of the patients had a history of chronic liver disease prior to surgery. Median duration of operation was significantly longer for patients with postoperative complications than those without. Furthermore, blood loss was significantly higher resulting in transfusion of red blood cells in 45% of patients with postoperative complications in comparison to only 14% in patients without complications. Child-Pugh score and histologically confirmed steatosis hepatis as well as preoperative biochemical liver values did not significantly differ between the two groups (Table 2).

**Table 2:** Comparison of characteristics between patients with and without complications

Variable		No Complication *N = 51*		Complication *N = 56*		*p*
		*N* or median	(%) or SD	*N* or median	(%) or SD	
Gender	Female	22	(43.14%)	15	(26.79%)	0.10^⋄^
	Male	29	(56.86%)	41	(73.21%)	
Age at operation		61.20	10.92	67.50	11.97	0.06^†^
BMI (kg/m^2^)		24.60	4.43	25.30	9.21	0.09^††^
ASA score	I	17	(33.33%)	20	(35.71%)	0.14^⋄^
	II	24	(47.06%)	17	(30.36%)	
	≥III	10	(19.61%)	19	(33.93%)	
Diabetes mellitus	Yes	7	(13.73%)	12	(21.43%)	0.32^⋄^
	No	44	(86.27%)	44	(78.57%)	
Cardiovascular disease	Yes	24	(47.06%)	25	(44.64%)	0.85^⋄^
	No	27	(52.94%)	31	(55.36%)	
Known Chronic liver disease	Yes	0	(0%)	0	(0%)	0.99^⋄^
	No	51	(100%)	56	(100%)	
Child-Pugh grade	A	51	(100%)	53	(94.64%)	0.25^⋄^
	B	0	(0%)	3	(5.36%)	
Steatosis	Yes	17	(33.33%)	18	(32.14%)	0.99^⋄^
	No	34	(66.67%)	38	(67.86%)	
Albumin (g/l)		43.90	4.40	43.00	5.31	0.13^††^
Bilirubin (mg/dl)		0.60	0.25	0.50	0.44	0.49^††^
Quick (%)		102.00	14.25	102.50	24.54	0.87^††^
Platelets (giga/l)		267.00	93.82	224.00	115.73	0.22*
NFS		-2.23	1.70	-0.82	1.79	<0.001^†^
FIB-4		1.23	0.72	1.81	0.92	0.002^††^
APRI		0.22	0.21	0.26	0.22	0.09^††^
SAFE		-2.36	104.39	102.97	114.73	<0.001^†^
T	1-2	7	(13.73%)	6	(10.71%)	0.76^⋄^
	3-4	44	(86.27%)	50	(89.29%)	
N	0	15	(29.41%)	22	(39.28%)	0.28^⋄^
	1	25	(49.02%)	19	(33.93%)	
	2	11	(21.57%)	15	(26.79%)	
Localization of metastases	Left lobe	8	(15.69%)	6	(10.71%)	0.71^⋄^
	Right lobe	15	(29.41%)	19	(33.93%)	
	Both lobes	28	(54.90%)	31	(55.36%)	
Neoadjuvant treatment	No	23	(45.10%)	32	(57.14%)	0.45^⋄^
	Chemotherapy	24	(47.06%)	20	(35.72%)	
	Radiochemotherapy	4	(7.84%)	4	(7.14%)	
Type of liver resection	Minor	39	(76.47%)	38	(67.86%)	0.39^⋄^
	Major	12	(23.53%)	18	(32.14%)	
Surgical approach	Open	38	(74.51%)	45	(80.36%)	0.49^⋄^
	Laparoscopic	13	(25.49%)	11	(19.64%)	
Simultaneous resection of primary tumor	Yes	18	(35.29%)	26	(46.43%)	0.32^⋄^
	No	33	(64.71%)	30	(53.57%)	
Length of surgery (min)		210.00	75.99	268	152.79	< 0.01^††^
Intraoperative blood loss (ml)		500.00	486.25	615.00	790.46	< 0.01^††^
Intraoperative blood transfusion	Yes	7	(13.73%)	25	(44.64%)	0.0006^⋄^
	No	44	(86.27%)	31	(55.36%)	

Data reported as n (%) or median with standard deviation. ^⋄^Statistical analysis by χ^2^ or Fishers exact test (in the case of ≤ 2 outcomes). ^†^Statistical analysis by Students *t*-test. ^††^Statistical analysis by Mann-Whitney *U* test. *ASA* American Society of Anesthesiologists, *APRI* AST-Platelet Ratio-index; *BMI* body mass index; *FIB-4* Fibrosis-4 Index 4; *NFS* NAFLD fibrosis score; *SAFE* steatosis-associated fibrosis estimator score

**Fig. 2:**
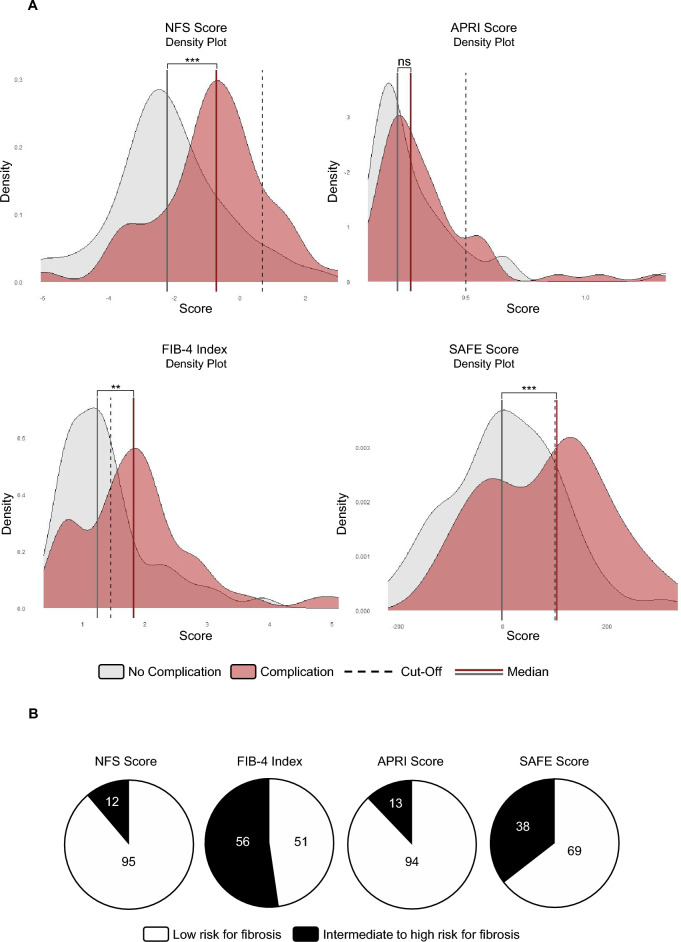
FIB-4 index and SAFE score correlate with postoperative complications after CRLM resection. **A** Density plot displaying the distribution of NFS, APRI, FIB-4, or SAFE values in patients with (red curve) or without (grey curve) postoperative complications. The dotted line represents the respective cut-off (NFS ≥ 0.675, APRI ≥ 0.5, FIB-4 ≥ 1.45, SAFE ≥ 100) indicating an increased risk for LF (*n* = 107; **p < 0.01, ***p < 0.001; Mann-Whitney *U* test). **B** Pie charts showing the number of patients with (black) or without (white) liver fibrosis as defined by NFS, APRI score, FIB-4 index, or SAFE score

Stratification of the patient collective for an intermediate to high risk for LF utilizing established cutoff values of each NIT resulted in a high percentage of patients at risk for LF, when assessing the FIB-4 index (52.3%) or SAFE score (35.5%). In contrast, the minority of patients displayed an intermediate to high risk for LF when assessing the NFS (11.2%) or APRI (12.2%) score (Fig. 2A and B). Patients who developed postoperative complications displayed significantly higher NFS, FIB-4, SAFE, but not APRI values compared with patients without postoperative complications (Fig. 2A). In summary, among all NITs evaluated for identifying patients at risk for postoperative complications, FIB-4 index, NFS, and SAFE score showed better discriminatory performance compared with APRI score. However, only FIB-4 index and SAFE score were able to differentiate between patients with and without complications when values were above their respective cutoffs. As shown in Fig. 2A, these cutoffs lie approximately between the median values of the two groups (patients with and without complications), indicating that they effectively capture the threshold for the occurrence of postoperative complications. Although the presence of LF was not confirmed histologically, the fact that FIB-4 index and SAFE score above the established cutoffs were significantly associated with postoperative complications supports their usefulness and applicability for preoperative risk stratification for LF-attributable postoperative complications.

### Noninvasive Tests for LF Predict Postoperative Complications After CRLM Resection

**Fig. 3:**
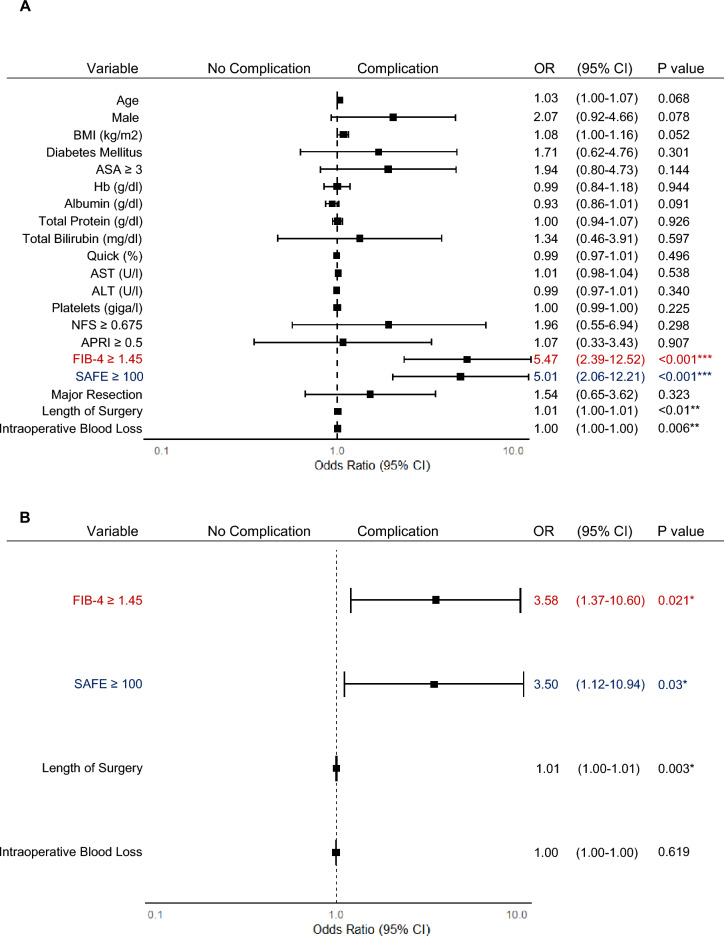
FIB-4 index and SAFE score are independent predictors of postoperative complications. **A** Univariate logistic regression analysis displaying the odds ratio (OR) with 95% confidence intervals (CI) for the occurrence of postoperative complications for each variable (**p < 0.01, ***p < 0.001). **B** Multivariate logistic regression analysis displaying the OR and 95% CI for the occurrence of postoperative complications using the variables length of surgery, intraoperative blood loss, and FIB-4 ≥ 1.45 or SAFE ≥ 100 respectively (*p < 0.05)

Preoperative liver (biochemical) values, including albumin, total bilirubin, and quick value as well as NFS and APRI score, did not correlate with the occurrence of postoperative complications. Baseline characteristics that were associated with postoperative complications included high risk of LF, as defined by FIB-4 index (odds ratio [OR] 5.47, 95% confidence interval [CI] 2.39–12.52; *p* < 0.001) or SAFE score (OR 5.01, 95% CI 2.06–12.21; *p* < 0.001) (Fig. 3A). Although statistically significant, length of surgery (OR 1.01, 95% CI 0.65–3.62; *p* < 0.01) and the amount of intraoperative blood loss (OR 1.00, 95% CI 1.00–1.00;* p* < 0.006) showed only a weak correlation with the development of postoperative complications (Fig. 3A). Multivariate analysis confirmed that FIB-4 index (OR 3.58, 95% CI 1.37–10.60;* p* < 0.021) and SAFE score (OR 3.50, 95% CI 1.12–10.94;* p* < 0.03) are independent predictors of postoperative complications (Fig. 3B). Consequently, further investigations on the impact of LF determined by NITs were focused solely on FIB-4 index and SAFE score.

### Liver Fibrosis as Defined by FIB-4 Index and SAFE Score Correlates with Surgical and Nonsurgical Postoperative Complications

Baseline characteristics after stratification for LF using FIB-4 index and SAFE score are shown in Supplement Tables 1 and 2. Patients stratified as intermediate to high risk for LF, either determined by FIB-4 index or SAFE score, had a higher median age compared with patients stratified as low risk for LF (FIB-4: 69.97 ± 10.98 vs. 59.63 ± 10.81; *p* < 0.001 and SAFE: 71.40 ± 10.74 vs. 60.94 ± 11.38; *p* < 0.001). The proportion of patients with type 2 diabetes mellitus was higher in the intermediate- to high-risk group compared with the low-risk group. In contrast, only patients with a positive FIB-4 index (but not SAFE score) were significantly more morbid, as defined by an ASA score ≥3 and had a higher intraoperative blood loss (Supplement Tables 1 and 2).

An intermediate to high risk for LF, indicated by FIB-4 index or SAFE score, correlated with the proportion and severity of postoperative complications (FIB-4: 22.64 ± 27.25 vs. 0.00 ± 20.46; *p* < 0.0001 and SAFE: 25.98 ± 27.36 vs. 0.00 ± 22.37; *p* < 0.001) compared with a low risk for LF (Tables 3 and 4). While an increased FIB-4 index was associated with the occurrence of a biliary leakage, only SAFE score (but not FIB-4 index) correlated with posthepatectomy haemorrhage and posthepatectomy liver failure (Tables 3 and 4). Both scores correlated with the occurrence of acute kidney injury (AKI), pleural effusion, and pneumonia (Tables 3 and 4). Patients with an intermediate to high risk for LF (independent of the investigated scores and indices) did not develop more wound dehiscence or cardiac arrhythmias. Patients with increased FIB-4 index or SAFE score displayed a more pronounced postoperative decline in liver function compared with those with lower scores (Supplement Tables 8 and 9), supporting the assumption that preoperatively high NITs correlate with impaired hepatic reserve.

**Table 3:** Postoperative complications in patients undergoing surgery for CRLM stratified for liver fibrosis by FIB-4 index

Variable		FIB-4 < 1.45 *N = 51*		FIB-4 ≥ 1.45 *N = 56*		*p*
		*N* or median	(%) or (SD)	*N* or median	(%) or (SD)	
Patients with postoperative complications	Yes	16	(31.37%)	40	(71.43%)	<0.0001^⋄^
	No	35	(68.63%)	16	(28.57%)	
Patients with complications according to Clavien-Dindo classification	I	3	(5.88%)	19	(33.93%)	<0.001^⋄^
	II	11	(21.56%)	25	(44.64%)	<0.05^⋄^
	IIIa	7	(13.73%)	13	(23.21%)	0.23^⋄^
	IIIb	4	(7.84%)	9	(16.07%)	0.24^⋄^
	IVa	0	(0.00%)	7	(12.50%)	<0.05^⋄^
	IVb	1	(1.96%)	2	(3.57%)	>0.99^⋄^
	V (mortality)	0	(0.00%)	4	(7.14%)	0.12^⋄^
CCI		0	20.46	22.64	27.25	<0.0001^††^
Biliary leakage		0	(0%)	9	(16.07%)	<0.01^⋄^
PHH		2	(3.92%)	8	(14.29%)	0.10^⋄^
PHLF		3	(5.88%)	7	(12.50%)	0.17^⋄^
Acute kidney injury		5	(9.80%)	15	(26.79%)	<0.05^⋄^
SSI	Superficial	1	(1.96%)	6	(10.71%)	<0.16^⋄^
	Deep	1	(1.96%)	4	(7.14%)	
	Organ/space	2	(3.92%)	2	(3.57%)	
Wound dehiscence		0	(0%)	2	(3.57%)	0.50^⋄^
Anastomotic leakage		2	(3.92%)	4	(7.14%)	0.68^⋄^
Ileus		9	(17.65%)	20	(35.71%)	<0.05^⋄^
Cardiac arrhythmia		1	(1.96%)	2	(3.57%)	>0.99^⋄^
Pleural effusion		5	(9.80%)	17	(30.36%)	<0.01^⋄^
Pneumonia		2	(3.92%)	10	(17.86%)	<0.05^⋄^
Urinary tract infection		1	(1.96%)	6	(10.71%)	0.12^⋄^

Data reported as n (%) or median with standard deviation. ^⋄^Statistical analysis by χ^2^ or Fishers exact test (in the case of ≤ 2 outcomes). ^††^Statistical analysis by Mann-Whitney *U* test. *CCI* comprehensive complication index; *FIB-4* Fibrosis-4 Index; *PHH* posthepatectomy hemorrhage; *PHLF* posthepatectomy liver failure; *SSI* surgical site infection

**Table 4:** Postoperative complications in patients undergoing surgery for CRLM stratified for liver fibrosis by SAFE score

Variable		SAFE < 100 *N = 69*		SAFE ≥ 100 *N = 38*		*p*
		*N* or median	(%) or (SD)	*N* or median	(%) or (SD)	
Patients with postoperative complications	Yes	27	(39.13%)	29	(76.32%)	< 0.001^⋄^
	No	42	(60.87%)	9	(23.68%)	
Patients with complications according to Clavien-Dindo classification	I	8	(11.59%)	14	(36.84%)	< 0.001^⋄^
	II	17	(24.64%)	19	(50.00%)	< 0.05^⋄^
	IIIa	9	(13.04%)	11	(28.95%)	0.07^⋄^
	IIIb	7	(10.15%)	6	(15.79%)	0.54^⋄^
	IVa	2	(2.90%)	5	(13.16%)	0.09^⋄^
	IVb	1	(1.45%)	2	(5.26%)	0.29^⋄^
	V (mortality)	1	(1.45%)	3	(7.89%)	0.13^⋄^
CCI		0.00	22.37	25.98	27.36	< 0.001^††^
Biliary leakage		4	(5.80%)	5	(13.16%)	0.28^⋄^
PHH		3	(4.35%)	7	(18.42%)	< 0.05^⋄^
PHLF		3	(4.35%)	7	(18.42%)	< 0.05^⋄^
Acute kidney injury		6	(8.70%)	14	(36.84%)	< 0.001^⋄^
SSI	Superficial	4	(5.80%)	3	(7.90%)	0.35^⋄^
	Deep	1	(1.45%)	4	(10.53%)	
	Organ/space	3	(4.35%)	1	(2.63%)	
Wound dehiscence		1	(1.45%)	1	(2.63%)	> 0.99^⋄^
Anastomotic leakage		4	(5.80%)	2	(5.26%)	> 0.99^⋄^
Ileus		13	(18.84%)	16	(42.11%)	0.01^⋄^
Cardiac arrhythmia		1	(1.45%)	2	(5.26%)	0.29^⋄^
Pleural effusion		8	(11.59%)	14	(36.84%)	< 0.01^⋄^
Pneumonia		3	(4.35%)	9	(23.68%)	< 0.01^⋄^
Urinary tract infection		2	(2.90%)	5	(13.16%)	0.09^⋄^

Data reported as n (%) or median with standard deviation. ^⋄^Statistical analysis by χ^2^ or Fishers exact test (in the case of ≤ 2 outcomes). ^††^Statistical analysis by Mann-Whitney *U* test. *CCI* comprehensive complication index; *PHH* posthepatectomy hemorrhage; *PHLF* posthepatectomy liver failure; *SSI* surgical site infection; *SAFE* steatosis-associated fibrosis estimator score

Intermediate to high risk for LF, as defined by SAFE score, was associated with a prolonged hospital and ICU stay as well as ICU readmission (Supplement Tables 3 and 4). FIB-4 index was associated with elevated rates of hospital readmission and length of stay on ICU (Supplement Tables 3 and 4), overall confirming that the short-term outcome of patients with a high risk for LF is impaired in our cohort.

### Validation of NITs in an Independent Second Cohort of Patients who Underwent Surgery for CRLM

To validate the predictive value of NITs for postoperative complications, we recapitulated key findings in an independent historic patient cohort (N = 277) who underwent liver resection of CRLM at the Department of General, Visceral, and Transplantation Surgery of the University Hospital Heidelberg. Baseline characteristics of this second cohort (Supplement Table 5) were comparable to those of the first cohort (Supplement Table 6), with similar median age, body mass index, and proportion of male patients. Patients in the second cohort showed more comorbidities, as indicated by a higher proportion of patients with ASA ≥ 3 score but displayed significantly fewer cases of type 2 diabetes mellitus (Supplement Table 6).

**Fig. 4:**
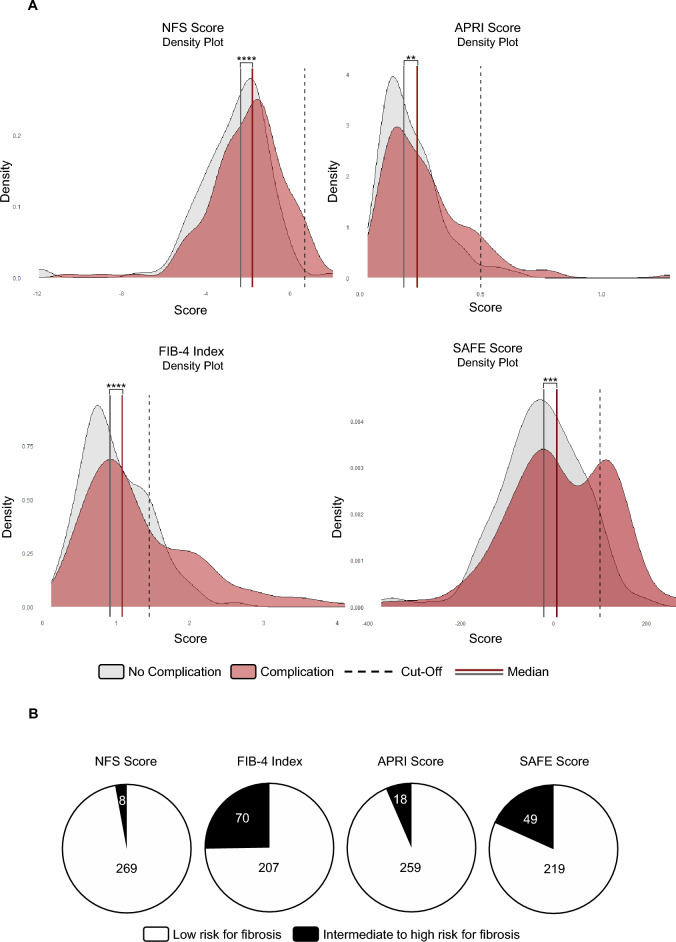
FIB-4 index and SAFE correlate with postoperative complications after CRLM resection in an independent second cohort. **A** Density plot displaying the distribution of NFS, APRI, FIB-4, or SAFE values in patients with (red curve) or without (grey curve) postoperative complications. The dotted line represents the respective cut-off (NFS ≥ 0.675, APRI ≥ 0.5, FIB-4 ≥ 1.45) indicating an elevated risk for LF (*n* = 277; **p < 0.01, ***p < 0.001, ****p < 0.0001; Mann-Whitney *U* test). **B** Pie charts showing the number of patients with (black) or without (white) LF as defined by the NFS, APRI score, FIB-4 index, or SAFE score

**Fig. 5:**
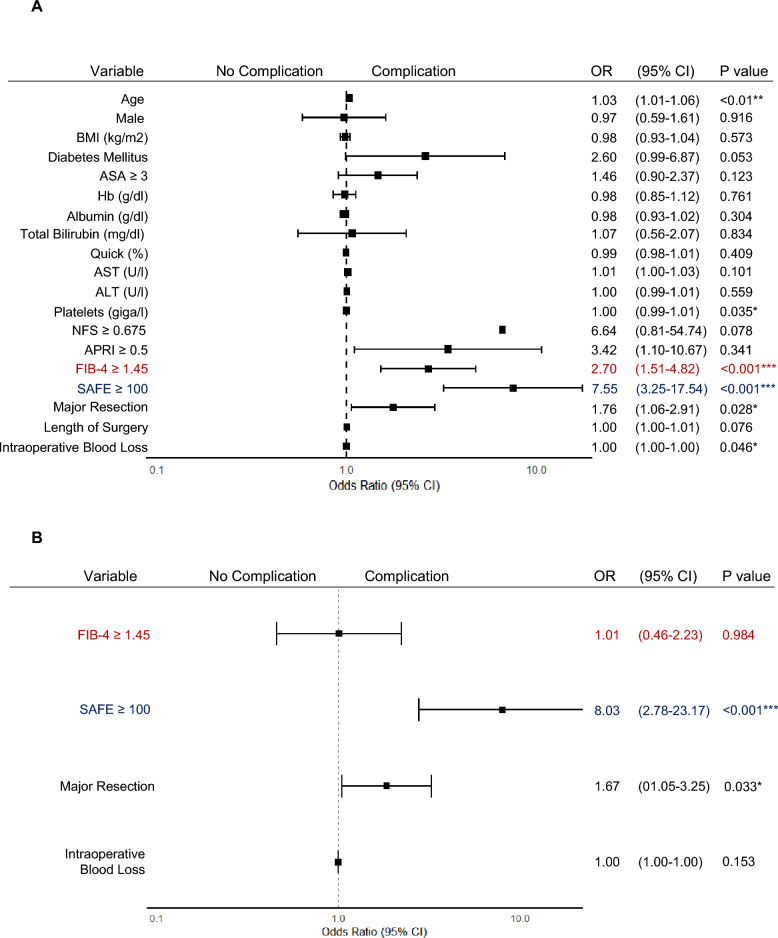
SAFE score is an independent predictor of postoperative complications in an independent second cohort. **A** Univariate logistic regression analysis displaying the odds ratio (OR) with 95% confidence intervals (CI) for the occurrence of postoperative complications for each variable (*p < 0.05, ***p < 0.001). **B** Multivariate logistic regression analysis displaying the OR and 95% CI for the occurrence of postoperative complications by using the variables major resection, blood loss, FIB-4 ≥ 1.45, and SAFE ≥ 100 (***p < 0.001). ^⋄^Bar for confidence intervals not depicted

In the second cohort, postoperative complications occurred in 52% of patients (Supplement Table 5). The median value of NFS was significantly higher in patients with postoperative complications confirming the findings from the first cohort (Fig. 4A). However, only stratification by FIB-4 index and SAFE score resulted in a large proportion of patients above the respective cutoff thresholds implying an intermediate to high risk for LF (Fig. 4B). As a result, FIB-4 index and SAFE score identified a significantly increased rate of postoperative complications for patients at risk for LF (Supplement Table 7). Uni- and multivariate logistic regression analyses confirmed that a high SAFE score (OR 8.03, 95% CI 2.78–23.17; *p* < 0.001) was an independent predictor of postoperative complications after adjusting for the confounding variables major resection and blood loss, reinforcing its utility in stratifying patients at risk for postoperative morbidity (Fig. 5). The reproducibility of the outlined findings across two independent patient cohorts supports the robustness of NITs, especially SAFE score, as preoperative tools to identify patients at risk for postoperative complications after resection of CRLM.

## Discussion

This study investigated the predictive significance of NITs to identify patients at risk for postoperative complications after CRLM resection. While NFS and APRI score were not associated with postoperative complications, FIB-4 index and SAFE score were independent risk factors for postoperative complications. Our findings were confirmed for SAFE score in an independent second cohort.

The comparatively high overall complication rate in our cohorts may be attributed to the inclusion of an elderly and multimorbid patient population, which is typical for CRLM surgery at tertiary centers. In addition, our comprehensive and systematic documentation of all postoperative events, including minor complications, may contribute to a higher overall rate compared with studies reporting only major complications. Importantly, the rate of major complications was within the range reported in previous studies.^15,17^ The validation of our findings in a large independent external cohort from a high-volume hepatobiliary center further supports the robustness and generalizability of our results.

Established risk factors for postoperative complications include patient related factors, such as frailty,^66,74^ multimorbidity,^52^ as well as prolonged length of surgery and elevated blood loss,^11^ suggesting extended resection or intraoperative complications. While end-stage liver disease is a well-established risk factor for surgical morbidity and mortality,^8,12^ subclinical LF is rarely considered in preoperative risk models for CRLM surgery. One of the underlying reasons is that preoperative assessment of LF remains challenging, because transdermal biopsy is seldom performed and subcirrhotic stages of LF often go undetected by clinical scores, such as Child-Pugh or MELD score.^53^ As a result of the low sensitivity for LF offered by Child-Pugh or MELD score, risk stratification in noncirrhotic patients seems futile. This notion is supported by the fact that they are not recommend for screening of LF^6^ and are barely associated with postoperative complications and mortality in noncirrhotic patients.^67,70,73^ In our study, 97.2% of patients were classified as Child-Pugh score A (Table 1). Because most patients were defined as Child-Pugh score A, correlative investigations between Child-Pugh score with the occurrence of postoperative complications (Table 2) and between Child-Pugh score and FIB-4 index or SAFE score was futile (Tables 3 and 4). While scores, such as the nontumor-related risk (NTRS) and ASA score represents an established preoperative evaluation tool, which are associated with long-term survival in CRC,^37,54^ they account more for the patients overall health status and presence of comorbidities rather than being a score to predict the presence of LF and LF-attributable risk for postoperative complications. However, previous studies showed an association between an elevated ASA score ≥ 3 and the occurrence of postoperative complications,^32^ as well as a correlation with increased FIB-4 values.^71^ We neither observed a significant correlation of ASA score ≥ 3 and FIB-4 index above the cutoff, nor with the occurrence of postoperative complications (Table 2). Taken together, our findings underscore the limited sensitivity of Child-Pugh and ASA scores and their restricted utility in a selected patient collective to screen for LF and predict postoperative complications. Notably, the Child-Pugh score was originally developed to estimate the prognosis and survival of patients with liver cirrhosis, particularly with regard to perioperative mortality in major surgical procedures. However, established liver cirrhosis was extremely rare in our cohort. In contrast, NITs, such as FIB-4 index and SAFE score that may detect subclinical LF that is otherwise missed during routine assessment correlated with the occurrence of postoperative complications and thus may improve preoperative risk stratification and prediction of postoperative morbidity. More specifically, higher FIB-4 and SAFE values were associated with an increased occurrence of postoperative AKI. Postoperative systemic inflammation and endothelial dysfunction are well-described risk factors for postoperative AKI^1,34^ and aggravated in patients with histologically confirmed LF.^48,61^ Previous studies have linked an elevated FIB-4 index with an increased rate of AKI in the general population^55^ as well as an impaired outcome in patients with chronic kidney disease.^14^ Our findings further promote the notion of the negative impact LF has on the development of AKI and the utility of NITs to stratify the risk of the development of AKI.

Furthermore, the correlation of LF and AKI explains the increased incidence of pleural effusion and pneumonia in patients with an increased FIB-4 index or SAFE score. Intriguingly, only stratification of the patient collective for LF by SAFE score correlated with the occurrence of posthepatectomy haemorrhage and posthepatectomy liver failure. In our cohort, complications occurred more frequently in patients with liver fibrosis (LF) identified by SAFE score, compared to those identified by FIB-4 index (Tables 3 and 4). This discrepancy was also observed in short-term outcome, because patients with a high risk for LF (detected by SAFE score) had significantly longer ICU and hospital stays compared with patients with a low risk for LF (detected by SAFE score), which was not observed when LF risk was determined by FIB-4 index. While both NITs (SAFE score and FIB-4 index) correlated with postoperative complications, SAFE score more accurately predicted short-term outcome compared to FIB-4 index. Previously, SAFE score offered good diagnostic accuracy in detecting fibrosis but was highly dependent on age.^68^ However, age alone did not appear to have a significant influence on the outcome in our cohort.

This study has several limitations: The relatively small sample size of the primary cohort represents a limitation of our study and may affect statistical power and precision of the estimated effect sizes. As such, the modest number of cases may limit generalizability. Larger multicenter or prospective studies are warranted to confirm our findings and to refine the predictive accuracy of non-invasive fibrosis scores in the perioperative setting. As histological evaluation of LF was not routinely performed in patients, correlation of an elevated NIT with histological presence of LF was not possible. We thus are unable to confirm that patients with high NIT scores truly display LF histologically. In general, elevated NITs might also be caused by frailty, malnutrition, and comorbidities leading to liver damage. Therefore, our observed strong correlation of elevated NITs with postoperative complications could likewise reflect an association with above-mentioned known risk factors.

In summary, an elevated risk for LF, as indicated by increased FIB-4 or SAFE values, is associated with postoperative complications after CRLM resection. Thus, preoperative assessment of FIB-4 index and SAFE score might be helpful to identify patients with CRLM who are at risk for postoperative complications after liver resection.

## Conclusions

FIB-4 index and SAFE score predict postoperative complications after liver resection due to CRLM. Prospective clinical studies analyzing the diagnostic accuracy and predictive value of NITs by utilizing liver specimen could further substantiate the clinical significance of preoperative NIT assessment in patients with CRLM.

## Supplementary Information

Below is the link to the electronic supplementary material.

Supplementary file1 (DOCX 85 KB)
