# Supplementary material for: Noninvasive Tests for Liver Fibrosis Predict Postoperative Complications After Resection of Colorectal Liver Metastases
Source: Ann Surg Oncol. 2026 Mar 25;33(7):6013–29. doi: 10.1245/s10434-026-19338-1 (PMC13242414; doi:10.1245/s10434-026-19338-1)
Supplement: Supplementary file 1 — Supplementary file1 (DOCX 85 KB) [file 10434_2026_19338_MOESM1_ESM.docx]

| Variable | | FIB-4 <1.45  *N = 51* | | FIB-4 ≥1.45  *N = 56* | | *p* |
| --- | --- | --- | --- | --- | --- | --- |
|  |  | *N* or median | (%) or SD | *N* or median | (%) or SD |  |
| Gender | Female | 21 | (41.18%) | 16 | (28.57%) | 0.22^◊^ |
|  | Male | 30 | (58.82%) | 40 | (71.43%) |  |
| Age at operation |  | 59.63 | 10.81 | 69.97 | 10.98 | <0.001^†^ |
| BMI (kg/m^2^) |  | 24.48 | 8.32 | 25.67 | 6.50 | <0.05^††^ |
| ASA score | I | 13 | (25.49%) | 24 | (42.86%) | <0.05^◊^ |
|  | II | 26 | (50.98%) | 15 | (26.78%) |  |
|  | ≥III | 12 | (23.53%) | 17 | (30.36%) |  |
| Diabetes mellitus | Yes | 5 | (9.80%) | 14 | (25.00%) | <0.05^◊^ |
|  | No | 46 | (90.20%) | 42 | (75.00%) |  |
| Cardio  -vascular disease | Yes | 23 | (45.10%) | 26 | (46.43%) | >0.99^◊^ |
|  | No | 28 | (54.90%) | 30 | (53.57%) |  |
| Known  chronic liver disease | Yes | 0 | (0%) | 0 | (0%) | >0.99^◊^ |
|  | No | 51 | (100%) | 56 | (100%) |  |
| Child-Pugh Grade | A | 50 | (98.04%) | 54 | (96.43%) | >0.99^◊^ |
|  | B | 1 | (1.96%) | 2 | (3.57%) |  |
| Steatosis | Yes | 17 | (33.3%) | 18 | (32.14%) | >0.99^◊^ |
|  | No | 34 | (67.7%) | 38 | (67.86%) |  |
| Albumin (g/l) |  | 43.60 | 5.24 | 43.75 | 4.69 | 0.95^††^ |
| Bilirubin (mg/dl) |  | 0.50 | 0.37 | 0.60 | 0.35 | 0.24^††^ |
| Quick (%) |  | 103.00 | 20.95 | 101.00 | 19.75 | 0.85^††^ |
| Platelets (giga/l) |  | 294.00 | 116.65 | 207.50 | 68.43 | <0.0001^††^ |
| NFS |  | -2.79 | 1.63 | -0.50 | 1.15 | <0.0001^††^ |
| FIB-4 |  | 0.88 | 0.28 | 2.00 | 0.77 | <0.0001^††^ |
| APRI |  | 0.18 | 0.10 | 0.33 | 0.25 | <0.0001^††^ |
| SAFE |  | -33.58 | 92.48 | 113.96 | 86.12 | <0.0001^††^ |
| T | 1-2 | 7 | (13.73%) | 6 | (10.71%) | 0.77^◊^ |
|  | 3-4 | 44 | (86.27%) | 50 | (89.29%) |  |
| N | 0 | 18 | (35.29%) | 19 | (33.93%) | 0.53^◊^ |
|  | 1 | 23 | (45.10%) | 21 | (37.5%) |  |
|  | 2 | 10 | (19.61%) | 16 | (28.57%) |  |
| Localization of metastases | Left lobe | 6 | (11.77%) | 8 | (14.28%) | 0.9^◊^ |
|  | Right lobe | 17 | (33.33%) | 17 | (30.36%) |  |
|  | Both lobes | 28 | (54.90%) | 31 | (55.36%) |  |
| Neoadjuvant treatment | No | 25 | (49.02%) | 30 | (53.57%) | 0.67^◊^ |
|  | Chemotherapy | 21 | (41.18%) | 23 | (41.07%) |  |
|  | Radio  -chemo  -therapy | 5 | (9.80%) | 3 | (5.36%) |  |
| Type of liver resection | Minor | 39 | (76.47%) | 38 | (67.86%) | 0.39^◊^ |
|  | Major | 12 | (23.53%) | 18 | (32.14%) |  |
| Surgical approach | Open | 39 | (76.47%) | 44 | (78.57%) | 0.82^◊^ |
|  | Laparo  -scopic | 12 | (23.53%) | 12 | (21.43%) |  |
| Resection of primary tumor | Yes | 22 | (43.14%) | 22 | (39.28%) | 0.08^◊^ |
|  | No | 29 | (56.86%) | 34 | (60.72%) |  |
| Length of surgery (min) |  | 236.00 | 129.04 | 228.00 | 130.37 | 0.79^††^ |
| Intra  -operative blood loss (ml) |  | 500.00 | 516.11 | 605.00 | 794.90 | <0.05^††^ |
| Intra  -operative blood transfusion | Yes | 14 | (27.45%) | 18 | (32.14%) | 0.67^◊^ |
|  | No | 37 | (72.55%) | 38 | (67.86%) |  |

**Supplement Table 1.** Comparison of baseline characteristics between patients with or without intermediate to high risk for liver fibrosis as defined by FIB-4 index. Data reported as n (%) or median with standard deviation. ^◊^Statistical analysis by Chi-X^2^ or Fisher´s exact test (in the case of ≤ 2 outcomes). ^†^Statistical analysis by Students *t*-test. ^††^Statistical analysis by Mann-Whitney U test. *ASA* American Society of Anesthesiologists, *APRI* AST-Platelet Ratio-index, *BMI* Body-mass-index, *FIB-4* Fibrosis-4 Index, *NFS* NAFLD fibrosis score, *SAFE* Steatosis-associated Fibrosis Estimator Score*.*

| Variable | | SAFE <100  *N = 69* | | SAFE ≥100  *N = 38* | | *p* |
| --- | --- | --- | --- | --- | --- | --- |
|  |  | *N* or median | (%) or SD | *N* or median | (%) or SD |  |
| Gender | Female | 27 | (39.13%) | 10 | (26.32%) | 0.21^◊^ |
|  | Male | 42 | (60.87%) | 28 | (73.68%) |  |
| Age at operation |  | 60.94 | 11.38 | 71.40 | 10.74 | <0.001^††^ |
| BMI (kg/m^2^) |  | 24.33 | 4.06 | 27.99 | 10.11 | <0.0001^††^ |
| ASA score | I | 25 | (36.23%) | 12 | (31.58%) | 0.09^◊^ |
|  | II | 30 | (43.48%) | 11 | (28.95%) |  |
|  | ≥III | 14 | (20.29%) | 15 | (39.47%) |  |
| Diabetes mellitus | Yes | 7 | (10.15%) | 12 | (31.6%) | <0.01^◊^ |
|  | No | 62 | (89.85%) | 26 | (68.4%) |  |
| Cardio  -vascular disease | Yes | 31 | (44.93%) | 18 | (47.37%) | 0.84^◊^ |
|  | No | 38 | (55.07%) | 20 | (52.63%) |  |
| Known  chronic liver disease | Yes | 0 | (0%) | 0 | (0%) | >0.99^◊^ |
|  | No | 69 | (100%) | 38 | (100%) |  |
| Child-Pugh grade | A | 68 | (98.55%) | 36 | (94.74%) | 0.29^◊^ |
|  | B | 1 | (1.45%) | 2 | (5.26%) |  |
| Steatosis | Yes | 22 | (31.88%) | 13 | (34.21%) | 0.83^◊^ |
|  | No | 47 | (68.12%) | 25 | (65.79%) |  |
| Albumin (g/l) |  | 43.80 | 4.50 | 42.90 | 5.44 | 0.10^††^ |
| Bilirubin (mg/dl) |  | 0.60 | 0.25 | 0.60 | 0.49 | 0.28^††^ |
| Quick (%) |  | 103.00 | 18.98 | 96.50 | 21.75 | 0.08^††^ |
| Platelets (giga/l) |  | 267.00 | 92.43 | 199.50 | 118.01 | <0.0001^††^ |
| NFS |  | -2.24 | 1.36 | -0.03 | 1.55 | <0.0001^††^ |
| FIB-4 |  | 1.19 | 0.45 | 2.16 | 0.86 | <0.0001^††^ |
| APRI |  | 0.20 | 0.11 | 0.39 | 0.27 | <0.0001^††^ |
| SAFE |  | -18.35 | 74.56 | 146.91 | 64.45 | <0.0001^††^ |
| T | 1-2 | 6 | (8.70%) | 7 | (18.42%) | 0.22^◊^ |
|  | 3-4 | 63 | (91.30%) | 31 | (81.58%) |  |
| N | 0 | 23 | (33.33%) | 14 | (36.84%) | 0.52^◊^ |
|  | 1 | 31 | (44.93%) | 13 | (34.21%) |  |
|  | 2 | 15 | (21.74%) | 11 | (28.95%) |  |
| Localization of metastases | Left lobe | 10 | (14.49%) | 4 | (10.53%) | 0.12^◊^ |
|  | Right lobe | 26 | (37.68%) | 8 | (21.05%) |  |
|  | Both lobes | 33 | (47.83%) | 26 | (68.42%) |  |
| Neoadjuvant treatment | No | 34 | (49.27%) | 21 | (55.27%) | 0.74^◊^ |
|  | Chemotherapy | 29 | (42.03%) | 15 | (39.47%) |  |
|  | Radio  -chemo  -therapy | 6 | (8.70%) | 2 | (5.26%) |  |
| Type of liver resection | Minor | 52 | (75.36%) | 25 | (65.79%) | 0.37^◊^ |
|  | Major | 17 | (24.64%) | 13 | (34.21%) |  |
| Surgical approach | Open | 53 | (76.81%) | 30 | (78.95%) | >0.99^◊^ |
|  | Laparo  -scopic | 16 | (23.19%) | 8 | (21.05%) |  |
| Resection of primary tumor | Yes | 29 | (42.03%) | 15 | (39.47%) | 0.84^◊^ |
|  | No | 40 | (58.97%) | 23 | (60.53%) |  |
| Length of surgery (min) |  | 240.00 | 137.15 | 220.00 | 114.40 | 0.55^††^ |
| Intra  -operative blood loss (ml) |  | 500.00 | 620.35 | 600.00 | 794.99 | 0.27^††^ |
| Intra  -operative blood transfusion | Yes | 18 | (26.09%) | 14 | (36.84%) | 0.28^◊^ |
|  | No | 51 | (73.91%) | 24 | (63.16%) |  |

**Supplement Table 2.** Comparison of baseline characteristics between patients with or without intermediate to high risk for liver fibrosis as defined by SAFE score. Data reported as n (%) or median with standard deviation. ^◊^Statistical analysis by Chi-X^2^ or Fisher´s exact test (in the case of < 2 outcomes). ^††^Statistical analysis by Mann-Whitney U test. *ASA* American Society of Anesthesiologists, *APRI* AST-Platelet Ratio-index, *BMI* Body-mass-index, *NFS* NAFLD fibrosis score, *FIB-4* Fibrosis-4 Index, *SAFE* Steatosis-associated Fibrosis Estimator Score*.*

| Variable | | FIB-4 <1.45  *N = 51* | | FIB-4 ≥1.45  *N = 56* | | *p* |
| --- | --- | --- | --- | --- | --- | --- |
|  |  | *N* or median | (%) or SD | *N* or median | (%) or SD |  |
| Length of hospital stay  (days) |  | 10.00 | 12.45 | 12.5 | 15.31 | 0.09^††^ |
| Length of ICU stay (days) |  | 1.00 | 8.25 | 2.00 | 13.09 | <0.001^††^ |
| Readmission to ICU | Yes | 0 | (0%) | 5 | (8.93%) | 0.06^◊^ |
|  | No | 51 | (100%) | 51 | (91.07%) |  |
| Readmission to hospital | Yes | 0 | (0%) | 9 | (16.07%) | <0.01^◊^ |
|  | No | 51 | (100%) | 47 | (83.93%) |  |

**Supplement Table 3.** Short-term outcome of patients with or without intermediate to high risk for liver fibrosis as defined by FIB-4 index. Data reported as n (%) or median with standard deviation. ^◊^Statistical analysis by Fisher´s exact test. ^††^Statistical analysis by Mann-Whitney U test. *FIB-4* Fibrosis-4 Index, *ICU* Intensive care unit.

| Variable | | SAFE <100  *N = 69* | | SAFE ≥100  *N = 38* | | *p* |
| --- | --- | --- | --- | --- | --- | --- |
|  |  | *N* or median | (%) or SD | *N* or median | (%) or SD |  |
| Length of hospital stay  (days) |  | 10.00 | 11.15 | 16.00 | 17.27 | <0.01^††^ |
| Length of ICU stay (days) |  | 1.00 | 7.38 | 2.00 | 15.41 | <0.001^††^ |
| Readmission to ICU | Yes | 0 | (0%) | 5 | (13.16%) | <0.01^◊^ |
|  | No | 69 | (100%) | 33 | (86.84%) |  |
| Readmission to hospital | Yes | 3 | (4.35%) | 6 | (15.79%) | 0.07^◊^ |
|  | No | 66 | (95.65%) | 32 | (84.21%) |  |

**Supplement Table 4.** Short-term outcome of patients with or without intermediate to high risk for liver fibrosis as defined by SAFE score. Data reported as n (%) or median with standard deviation. ^◊^Statistical analysis by Fisher´s exact test. ^††^Statistical analysis by Mann-Whitney U test. *SAFE* Steatosis-associated Fibrosis Estimator Score*, ICU* Intensive care unit.

| Variable | | *N* (= 277) or  median | (%) or SD |
| --- | --- | --- | --- |
| Gender | Female | 89 | (32.13%) |
|  | Male | 188 | (67.87%) |
| Age at operation |  | 63.00 | 10.41 |
| BMI (kg/m^2^) |  | 26.23 | 4.24 |
| ASA score | I | 7 | (2.53%) |
|  | II | 156 | (56.32%) |
|  | ≥III | 114 | (41.15%) |
| Diabetes mellitus | Yes | 22 | (7.94%) |
|  | No | 255 | (92.06%) |
| Cardiovascular disease | Yes | 66 | (23.83%) |
|  | No | 211 | (76.17%) |
| Known  chronic liver disease | Yes | 1 | (0.36%) |
|  | No | 276 | (99.64%) |
| Steatosis | Yes | 105 | (37.91%) |
|  | No | 87 | (31.41%) |
|  | Unknown | 85 | (30.68%) |
| Albumin (g/l) |  | 43.00 | 5.09 |
| Total bilirubin (mg/dl) |  | 0.50 | 0.66 |
| AST (U/l) |  | 22.00 | 14.85 |
| ALT(U/l) |  | 24.00 | 22.97 |
| Quick (%) |  | 103.00 | 14.54 |
| Platelets (giga/l) |  | 259.00 | 111.62 |
| NFS |  | -2.16 | 1.84 |
| APRI |  | 0.20 | 0.16 |
| FIB-4 |  | 1.01 | 0.66 |
| SAFE |  | -8.59 | 103.68 |
| T | 1-2 | 44 | (15.89%) |
|  | 3-4 | 229 | (82.67%) |
|  | Unknown | 4 | (1.44%) |
| N | 0 | 100 | (36.10%) |
|  | 1 | 88 | (31.77%) |
|  | 2 | 81 | (29.24%) |
|  | Unknown | 8 | (2.89%) |
| Localization of the primary tumor | Coecum  Colon ascendens  Colon transversum  Colon descendens  Colon sigmoideum  Rectum  Double carcinoma | 24  27  5  14  79  122  6 | (8.66%)  (9.75%)  (1.81%)  (5.05%)  (28.52%)  (44.04%)  (2.17%) |
|  |  |  |  |
| Number of infiltrated liver segments | 1  2  3  4  >5 | 70  78  50  36  43 | (25.27%)  (28.16%)  (18.05%)  (13.00%)  (15.52%) |
| Neoadjuvant treatment | No | 200 | (72.20%) |
|  | Chemotherapy | 77 | (27.80%) |
| Type of liver resection | Major | 184 | (66.43%) |
|  | Minor | 93 | (33.57%) |
| Number of resected segments | 1  2  3  4  >5 | 39  54  33  83  68 | (14.08%)  (19.50%)  (11.91%)  (29.96%)  (24.55%) |
| Time point of metastases | Synchronous | 111 | (40.07%) |
|  | Metachronous | 166 | (59.93%) |
| Length of surgery (min) |  | 200.00 | 83.98 |
| Intraoperative blood loss (ml) |  | 600.00 | 668.20 |
| Intraoperative blood transfusion | Yes | 66 | (23.83%) |
|  | No | 211 | (76.17%) |
| Patients with postoperative complications | Yes | 145 | (52.35%) |
|  | No | 132 | (47.65%) |
| Bile leakage | Yes | 59 | (21.30%) |
|  | No | 218 | (78.70%) |
| Thrombosis | Yes | 3 | (1.08%) |
|  | No | 274 | (98.92%) |
| Pulmonary embolism | Yes | 5 | (1.81%) |
|  | No | 272 | (98.19%) |
| PHH | Yes | 34 | (12.27%) |
|  | No | 243 | (87.73%) |
| PHLF | Yes | 20 | (7.22%) |
|  | No | 257 | (92.78%) |
| Cardiovascular | Yes | 6 | (2.17%) |
|  | No | 271 | (97.83%) |
| Pleural effusion / respiratory insufficiency | Yes | 36 | (13.00%) |
|  | No | 241 | (87.00%) |
| Postoperative infection | SSI | 10 | (3.61%) |
|  | Pneumonia | 16 | (5.78%) |
|  | Urinary tract infection | 36 | (13.00%) |

**Supplement Table 5.** Characteristics of all UICC stage 4 CRC patients from the 2^nd^ cohort. Data reported as n (%) or median with standard deviation. *ASA* American Society of Anesthesiologists, *AST* Aspartate-Amino-transferase, *ALT* Alanine-Aminotransferase, *APRI* AST-Platelet Ratio-index, *BMI* Body-mass-index, *FIB-4* Fibrosis-4 Index, *NFS* NAFLD fibrosis score, *SSI* Surgical site infection, *PHH* Post-hepatectomy hemorrhage, *PHLF* Post-hepatectomy liver failure, *SAFE* Steatosis-associated Fibrosis Estimator Score*.*

| Variable | | 1^st^ Cohort | | | 2^nd^ Cohort | | | *p* |
| --- | --- | --- | --- | --- | --- | --- | --- | --- |
|  |  | (N = 107) | | | (N = 277) | | |  |
|  |  | *N* or median | | (%) or SD | *N* or median | | (%) or SD |  |
| Patients with postoperative complications | Yes | 56 | (52.34%) | | 145 | (52.35%) | | >0.99^◊^ |
|  | No | 51 | (47.66%) | | 132 | (47.65%) | |  |
| Gender | Female | 37 | (34.58%) | | 89 | (32.13%) | | 0.71^◊^ |
|  | Male | 70 | (65.42%) | | 188 | (67.87%) | |  |
| Age at operation |  | 63.40 | 11.67 | | 63.00 | 10.41 | | 0.09^††^ |
| BMI (kg/m^2^) |  | 25.00 | 7.48 | | 26.23 | 4.24 | | 0.33^††^ |
| ASA | I-II | 78 | (72.90%) | | 163 | (58.84%) | | <0.01^◊^ |
|  | ≥III | 29 | (27.10%) | | 114 | (41.16%) | |  |
| Diabetes mellitus | Yes  No | 19  88 | (17.76%)  (82.24%) | | 22  255 | (7.94%)  (92.06%) | | <0.001^◊^ |
| Hb (g/dl) |  | 12.75 | 2.21 | | 13.5 | 1.72 | | <0.0001^†^ |
| Albumin (g/dl) |  | 43.60 | 4.96 | | 43.00 | 5.09 | | 0.66^††^ |
| Total Bilirubin (mg/dl) |  | 0.60 | 0.36 | | 0.50 | 0.66 | | 0.17^††^ |
| Quick (%) |  | 102 | 20.33 | | 103.00 | 14.54 | | 0.86^††^ |
| AST (U/l) |  | 25.00 | 13.91 | | 22.00 | 14.85 | | <0.001^††^ |
| ALT (U/l) |  | 21.00 | 19.38 | | 24.00 | 22.97 | | 0.03^††^ |
| Platelets (giga/l) |  | 252.00 | 91.54 | | 259.00 | 111.62 | | 0.07^††^ |
| NFS |  | -1.44 | 1.84 | | -2.16 | 1.84 | | <0.0001^††^ |
| APRI |  | 0.24 | 0.22 | | 0.20 | 0.16 | | <0.001^††^ |
| FIB-4 |  | 1.48 | 0.86 | | 1.01 | 0.66 | | <0.0001^††^ |
| SAFE |  | 38.85 | 116.63 | | -8.59 | 103.68 | | <0.001^††^ |
| Major liver resection | Yes | 30 | (28.04%) | | 184 | (66.43%) | | <0.0001^◊^ |
|  | No | 77 | (71.96%) | | 93 | (33.57%) | |  |
| Length of surgery (min) |  | 233.00 | 129.81 | | 200.00 | 83.98 | | <0.01** |
| Intraoperative blood loss (ml) |  | 600.00 | 692.48 | | 600.00 | 668.20 | | 0.22** |

**Supplement Table 6.** Comparative analysis of the 1^st^ and 2^nd^ cohort. Data reported as n (%) or median with standard deviation. ^◊^Statistical analysis by Fisher´s exact test. ^†^Statistical analysis by Students *t*-test. ^††^Statistical analysis by Mann-Whitney U test. *ASA* American Society of Anesthesiologists *AST* Aspartate-Amino-transferase, *ALT* Alanine-Aminotransferase, *APRI* AST-Platelet Ratio-index, *BMI* Body-mass-index, *FIB-4* Fibrosis-4 Index, *Hb* hemoglobin, *NFS* NAFLD fibrosis score, *SAFE* Steatosis-associated Fibrosis Estimator Score*.*

| Variable | | No Complication | (%) | Complication | (%) | *p* |
| --- | --- | --- | --- | --- | --- | --- |
|  |  | *N = 132* |  | *N = 145* |  |  |
| NFS | < 0.675 | 131 | (99.24%) | 138 | (95.17%) | 0.07^◊^ |
|  | ≥ 0.675 | 1 | (0.76%) | 7 | (4.83%) |  |
| APRI | < 0.5 | 128 | (96.97%) | 131 | (90.34%) | 0.02^◊^ |
|  | ≥ 0.5 | 4 | (3.03%) | 14 | (9.66%) |  |
| FIB-4 | < 1.45 | 111 | (84.10%) | 96 | (66.21%) | 0.0008^◊^ |
|  | ≥ 1.45 | 21 | (15.90%) | 49 | (33.79%) |  |
| SAFE | < 100 | 122 | (92.42%) | 97 | (66.90%) | <0.0001^◊^ |
|  | ≥ 100 | 7 | (5.30%) | 42 | (28.96%) |  |
|  | Unknown | 3 | (2.28%) | 6 | (4.14%) |  |

**Supplement Table 7.** Comparison of patient numbers in the 2^nd^ cohort with or without complications, stratified by the respective cut-off of each NIT. Data reported as n (%). ^◊^Statistical analysis by Chi-X^2^ or Fisher´s exact test (in the case of < 2 outcomes).  *APRI* AST-Platelet Ratio-index, *FIB-4* Fibrosis-4 Index 4, *NFS* NAFLD fibrosis score. *SAFE* Steatosis-associated Fibrosis Estimator Score*.*

| Variable | | FIB-4  < 1.45  *N = 51* | | FIB-4  ≥ 1.45  *N = 56* | | *p* |
| --- | --- | --- | --- | --- | --- | --- |
|  |  | Median | SD | Median | SD |  |
| Albumin | PreOP | 43.60 | 5.24 | 43.75 | 4.69 | >0.95^††^ |
|  | POD1 | 32.10 | 4.41 | 30.60 | 4.16 | 0.05^†^ |
|  | POD2 | 32.90 | 4.60 | 31.75 | 3.63 | 0.26^†^ |
|  | POD5 | 34.80 | 5.86 | 32.65 | 4.59 | 0.20^†^ |
|  | POD7 | 32.70 | 5.55 | 32.40 | 4.87 | 0.80^†^ |
| Bilirubin | PreOP | 0.50 | 0.37 | 0.60 | 0.35 | 0.51^††^ |
|  | POD1 | 0.90 | 0.85 | 0.10 | 1.24 | 0.07^††^ |
|  | POD2 | 0.60 | 0.86 | 0.85 | 1.31 | 0.01^††^ |
|  | POD5 | 0.50 | 0.48 | 0.80 | 1.20 | <0.01^††^ |
|  | POD7 | 0.40 | 0.67 | 0.70 | 1.26 | <0.05^††^ |
|  | POD9 | 0.45 | 0.37 | 0.80 | 2.11 | <0.05^††^ |
| Quick (%) | PreOP | 103.00 | 20.95 | 101.00 | 19.75 | 0.85^††^ |
|  | POD1 | 77.00 | 15.50 | 71.50 | 17.06 | 0.09^†^ |
|  | POD2 | 87.00 | 19.73 | 75.00 | 22.28 | 0.06^†^ |
|  | POD5 | 96.5 | 18.91 | 91.00 | 32.34 | 0.17^††^ |
|  | POD7 | 95.50 | 19.25 | 86.00 | 18.89 | 0.66^†^ |
|  | POD9 | 90 | 20.53 | 83 | 13.79 | 0.40^†^ |
| Platelets (giga/l) | PreOP | 294.00 | 116.65 | 207.50 | 68.43 | <0.0001^††^ |
|  | POD1 | 215.00 | 79.45 | 158.00 | 66.81 | <0.0001^††^ |
|  | POD2 | 198.50 | 86.92 | 217.50 | 88.84 | <0.001^††^ |
|  | POD5 | 270.50 | 119.40 | 185.00 | 96.02 | <0.001^††^ |
|  | POD7 | 260.50 | 114.90 | 221.00 | 114.60 | 0.08^††^ |
| AST (u/l) | PreOP | 21.00 | 12.81 | 28.50 | 14.19 | <0.001^††^ |
|  | POD1 | 175.00 | 538.50 | 314.50 | 363.60 | <0.05^††^ |
|  | POD2 | 81.00 | 224.10 | 408.50 | 230.70 | <0.05^††^ |
|  | POD5 | 41.00 | 32.51 | 57.00 | 47.34 | <0.01^††^ |
|  | POD7 | 40.00 | 30.36 | 46.00 | 29.89 | 0.20^††^ |
| ALT (u/l) | PreOP | 21.00 | 22.20 | 20.00 | 16.51 | 0.25^††^ |
|  | POD1 | 172.00 | 452.00 | 256.00 | 270.90 | 0.26^††^ |
|  | POD2 | 144.00 | 356.70 | 259.50 | 260.90 | 0.19^††^ |
|  | POD5 | 78.00 | 137.60 | 97.00 | 106.80 | 0.37^††^ |
|  | POD7 | 71.00 | 109.40 | 82.50 | 68.21 | 0.65^††^ |

**Supplement Table 8.** Comparison of postoperative liver values between patients with or without intermediate to high risk for liver fibrosis as defined by FIB-4 index. Data reported as median with standard deviation. ^†^Statistical analysis by Students *t*-test. ^††^Statistical analysis by Mann-Whitney U test. *AST* Aspartate-Amino-transferase, *ALT* Alanine-Aminotransferase, *FIB-4* Fibrosis-4 Index, *POD* Postoperative day.

| Variable | | SAFE  < 100  *N = 69* | | SAFE  ≥ 100  *N = 38* | | *p* |
| --- | --- | --- | --- | --- | --- | --- |
|  |  | Median | SD | Median | SD |  |
| Albumin | PreOP | 43.80 | 4.50 | 42.90 | 5.44 | 0.10^††^ |
|  | POD1 | 31.90 | 4.36 | 30.50 | 4.14 | 0.05^†^ |
|  | POD2 | 32.90 | 4.52 | 31.75 | 3.32 | 0.41^†^ |
|  | POD5 | 33.80 | 5.33 | 32.80 | 5.15 | 0.50^†^ |
|  | POD7 | 32.95 | 5.40 | 33.28 | 4.68 | 0.23^†^ |
| Bilirubin | PreOP | 0.60 | 0.25 | 0.60 | 0.49 | 0.28^††^ |
|  | POD1 | 0.90 | 1.07 | 1.10 | 1.10 | 0.27^††^ |
|  | POD2 | 0.70 | 1.17 | 0.85 | 1.08 | 0.18^††^ |
|  | POD5 | 0.50 | 0.94 | 0.80 | 0.98 | <0.05^††^ |
|  | POD7 | 0.50 | 1.26 | 0.70 | 0.66 | 0.19^††^ |
|  | POD9 | 0.50 | 2.12 | 0.85 | 0.55 | <0.05^††^ |
| Quick (%) | PreOP | 103.00 | 18.98 | 96.50 | 21.75 | 0.08^††^ |
|  | POD1 | 77.50 | 15.96 | 69.00 | 17.30 | 0.12^††^ |
|  | POD2 | 86.00 | 19.94 | 72.00 | 23.31 | 0.13^†^ |
|  | POD5 | 97.00 | 25.42 | 91.00 | 27.78 | 0.22^††^ |
|  | POD7 | 95.50 | 20.30 | 85.00 | 17.51 | 0.71^†^ |
|  | POD9 | 89.50 | 20.22 | 84.00 | 11.92 | 0.55^†^ |
| Platelets (giga/l) | PreOP | 267.00 | 92.43 | 199.50 | 118.01 | <0.0001^††^ |
|  | POD1 | 209.00 | 80.66 | 152.00 | 64.00 | <0.001^††^ |
|  | POD2 | 186.00 | 86.47 | 136.00 | 94.33 | <0.05^††^ |
|  | POD5 | 225.50 | 117.70 | 202.00 | 94.98 | <0.05^††^ |
|  | POD7 | 254.50 | 116.80 | 198.00 | 110.00 | 0.07^††^ |
| AST (u/l) | PreOP | 22.00 | 9.37 | 35.50 | 17.04 | <0.0001^††^ |
|  | POD1 | 241.50 | 487.20 | 272.00 | 385.00 | 0.38^††^ |
|  | POD2 | 104.00 | 232.20 | 169.00 | 229.30 | 0.25^††^ |
|  | POD5 | 43.50 | 46.72 | 41.50 | 32.90 | 0.07^††^ |
|  | POD7 | 38.50 | 30.74 | 37.50 | 29.25 | 0.15^††^ |
| ALT (u/l) | PreOP | 20.00 | 19.83 | 21.50 | 18.68 | 0.26^††^ |
|  | POD1 | 202.50 | 404.50 | 235.50 | 278.80 | 0.91^††^ |
|  | POD2 | 174.00 | 337.00 | 204.00 | 256.70 | 0.65^††^ |
|  | POD5 | 94.50 | 132.50 | 82.00 | 102.00 | 0.67^††^ |
|  | POD7 | 71.50 | 106.00 | 81.00 | 49.97 | 0.93^††^ |

**Supplement Table 9.** Comparison of postoperative liver values between patients with or without intermediate to high risk for liver fibrosis as defined by SAFE score. Data reported as median with standard deviation. ^†^Statistical analysis by Students *t*-test. ^††^Statistical analysis by Mann-Whitney U test. *AST* Aspartate-Amino-transferase, *ALT* Alanine-Aminotransferase, *POD* Postoperative day. *SAFE* Steatosis-associated Fibrosis Estimator Score*.*
